# WDR5, BRCA1, and BARD1 Co-regulate the DNA Damage Response and Modulate the Mesenchymal-to-Epithelial Transition during Early Reprogramming

**DOI:** 10.1016/j.stemcr.2019.02.006

**Published:** 2019-03-14

**Authors:** Georgina Peñalosa-Ruiz, Vicky Bousgouni, Jan P. Gerlach, Susan Waarlo, Joris V. van de Ven, Tim E. Veenstra, José C.R. Silva, Simon J. van Heeringen, Chris Bakal, Klaas W. Mulder, Gert Jan C. Veenstra

**Affiliations:** 1 Department of Molecular Developmental Biology, Faculty of Science, Radboud Institute for Molecular Life Sciences, Radboud University, Nijmegen 6500 HB, the Netherlands; 2 Dynamical Cell Systems Team, Division of Cancer Biology, Chester Beatty Laboratories Institute of Cancer Research, 237 Fulham Road, London SW3 6JB, UK; 3 Welcome Trust Medical Research Council Cambridge Stem Cell Institute and Department of Biochemistry, University of Cambridge, Tennis Court Road, Cambridge CB2 1QR, UK

**Keywords:** reprogramming, iPSCs, mesenchymal-to-epithelial transition, DNA damage repair, functional interactions, chromatin factors, WDR5, BRCA1, BARD1

## Abstract

Differentiated cells are epigenetically stable, but can be reprogrammed to pluripotency by expression of the OSKM transcription factors. Despite significant effort, relatively little is known about the cellular requirements for reprogramming and how they affect the properties of induced pluripotent stem cells. We have performed high-content screening with small interfering RNAs targeting 300 chromatin-associated factors and extracted colony-level quantitative features. This revealed five morphological phenotypes in early reprogramming, including one displaying large round colonies exhibiting an early block of reprogramming. Using RNA sequencing, we identified transcriptional changes associated with these phenotypes. Furthermore, double knockdown epistasis experiments revealed that BRCA1, BARD1, and WDR5 functionally interact and are required for the DNA damage response. In addition, the mesenchymal-to-epithelial transition is affected in *Brca1*, *Bard1*, and *Wdr5* knockdowns. Our data provide a resource of chromatin-associated factors in early reprogramming and underline colony morphology as an important high-dimensional readout for reprogramming quality.

## Highlights

• High-content imaging screen of chromatin factors in reprogramming MEFs to iPSCs
• Colony level phenotypes followed up with secondary screen using RNA sequencing
• Wdr5 functionally interacts with Brca1 and Bard1 in early reprogramming processes
• WDR5, BRCA1, and BARD1 affect DNA repair and mesenchymal-to-epithelial transition

## Graphical Abstract

**Figure:**
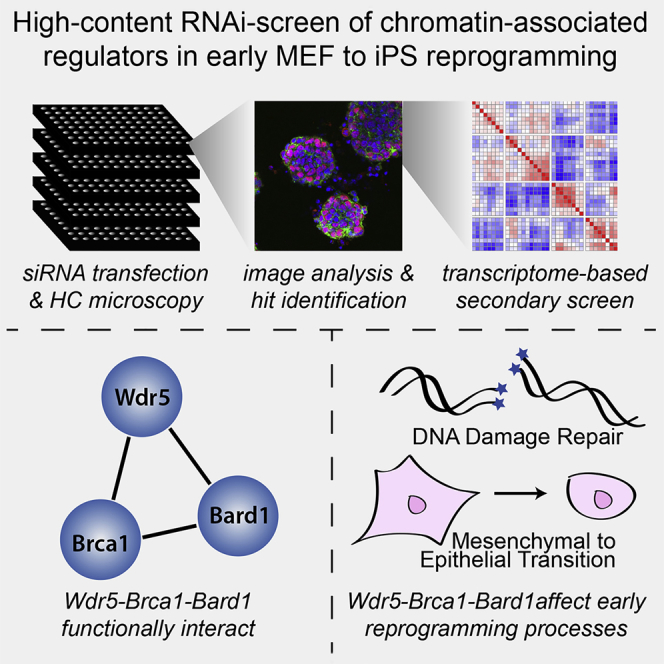

## Teaser

To assess the role of chromatin-associated factors in reprogramming of fibroblasts to induced pluripotency, high-content imaging screening with siRNAs targeting chromatin-associated factors was performed to identify colony level phenotypes. RNA sequencing was used as a secondary screen. It was found that WDR5 functionally interacts with BRCA1 and BARD1, and that these factors are important for DNA repair and the mesenchymal-to-epithelial transition.

## Introduction

Somatic cells can be reprogrammed to pluripotency by artificial expression of four transcription factors: OCT4, SOX2, KLF4, and c-MYC (OSKM) (Takahashi and Yamanaka, 2006). With varying efficiency, induced pluripotent stem cells (iPSCs) can be derived from a wide variety of cell types and they can differentiate into all cell lineages. Thus, they represent a promising resource for tissue regeneration and disease modeling.

Reprogramming occurs in two transcriptional and epigenetic waves (Polo et al., 2012). During the first wave, cell proliferation increases, and cells must overcome senescence and apoptosis (Xu et al., 2016). Dramatic metabolic changes and fast proliferation trigger the DNA damage response (DDR) through activation of the p53 pathway, inducing apoptosis on cells carrying substantial DNA damage (Marion et al., 2009). In agreement, DDR and DNA replication complexes are highly induced (Hansson et al., 2012), and DDR proteins, such as BRCA1, are required for efficient reprogramming (Gonzalez et al., 2013, Hansson et al., 2012). Senescence is also a barrier for reprogramming (Utikal et al., 2009). Some aging hallmarks, such as eroded telomeres (Lapasset et al., 2011, Marion and Blasco, 2010) and senescence-associated epigenetic marks (Ocampo et al., 2016) need to be reset by OSKM for efficient reprogramming. At the transcriptional level, the somatic program is silenced concurrent with the acquisition of epithelial characteristics (Li et al., 2010, Samavarchi-Tehrani et al., 2010). Transcriptional changes during these stages are quite heterogeneous, whereas the pluripotency program is activated in a sequential and hierarchical manner (Buganim et al., 2012). Some of the early upregulated pluripotency genes include *SSEA1*, and the epithelial genes *Cdh1* and *Epcam*, and *Sall4*. At this point, reprogramming intermediates have only partially acquired the pluripotency program (Silva et al., 2008). Most of the cells will be trapped in such stages and only a small proportion will progress toward full pluripotency (Polo et al., 2012). Reactivation of *Sox2* (Buganim et al., 2012), *Nanog*, and *Esrrb* occurs during the second transcriptional and epigenetic wave and is also rate limiting to complete reprogramming (Apostolou and Stadtfeld, 2018).

During these transcriptional waves, chromatin dynamics involves the interplay of chromatin modifiers, transcriptional regulators and OSKM binding activities. Initially, OSK bind to open enhancers in mouse embryonic fibroblasts (MEFs) and consequently co-repressors, such as NCoR/SMRT are recruited to silence somatic genes (Zhuang et al., 2018). Also during early phases, H3K4me2 is rapidly deposited at some pluripotency-associated loci (Xu et al., 2016). Accordingly, SET-MLL methyltransferase complexes, including their core component WDR5, have been shown to be crucial to facilitate reprogramming through H3K4me2/me3 deposition at pluripotency-associated regulatory regions (Ang et al., 2011, Wang et al., 2016). Other stem cell regulators reside within H3K9me3 heterochromatic domains (Apostolou and Stadtfeld, 2018). In concordance, activities of H3K9 methyl transferases EHMT1/2, SUV39H1/2, and SETDB1 constitute roadblocks of reprogramming (Soufi et al., 2012, Sridharan et al., 2013), whereas H3K9 demethylases such as KDM3A/B and KDM4C are facilitators (Chen et al., 2013). These and many other key chromatin regulators have been identified by RNAi (Cacchiarelli et al., 2015, Qin et al., 2014, Xu et al., 2016).

Despite the progress that has been made in characterizing the molecular changes during reprogramming, it is not fully understood how these dynamic changes are orchestrated. We have used high-content screening to assess the role of ∼300 chromatin-associated proteins in colony phenotypes during early reprogramming. The combination of small interfering RNA (siRNA) screening with high-content microscopy allows simultaneous measurement of multiple morphological phenotypes and can reveal new associations among pathways (Fischer et al., 2015, Sero and Bakal, 2017). A similar approach has previously been used to define new gene networks involved in the final phase of iPSC formation (Golipour et al., 2012). We measured more than 20 colony features, including number of colonies, expression of early pluripotency markers, and other morphological and texture features, after individual knockdown of 300 chromatin modifiers. Selected hits from the primary screening were subjected to a transcriptome-based secondary screen. We identify several chromatin-associated genes that act together in the DDR and the mesenchymal-to-epithelial transition (MET) during early reprogramming.

## Results

### High-Throughput Analysis of the Early Phase of Reprogramming

**Figure 1:**
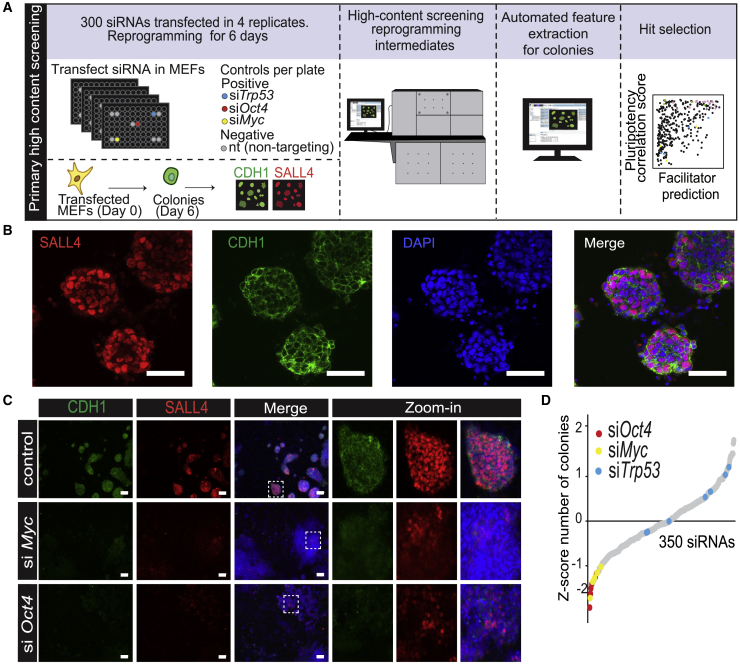
High-Throughput Analysis of the Early Phase of Reprogramming (A) Experimental design of high-content imaging siRNA screen. (B) Representative image showing an immunofluorescence of reprogramming intermediates at day 6 stained for pluripotency markers CDH1 and SALL4 with DAPI counterstain. Scale bars, 50 μm. (C) Comparison of colony phenotypes of control, si*Myc* and si*Oct4* cells at reprogramming day 6, stained for SALL4 and CDH1. Scale bars, 100 μm (images on the right, 4× zoom-in of inset). (D) siRNAs in the whole screen ranked from low to high *Z* scores, based on the number of colonies. Positive controls are highlighted in colors. Each siRNA represents the average *Z* score from four replicates (independent transfections in the same experiment). There are 350 siRNAs because controls are included in the rank. See also Figure S1 and Table S1. siRNA Library, Related to Figures 1 and 2, Table S2. Screening Complete Dataset, Related to Figure 2, Table S3. Screening Selected Features, Related to Figure 2.

Reprogramming is associated with major changes in cell morphology, in part due to the MET (Li et al., 2010). Thus, we asked whether chromatin-mediated changes would affect reprogramming efficiency, colony morphology, and expression of early pluripotency markers. Moreover, we wondered how chromatin-associated factors might work together, as revealed by their similarities in a high-dimensional phenotypic space upon knockdown (Mulder et al., 2012, Wang et al., 2012). To define a set of relevant chromatin-associated factors for an siRNA screen (Figure 1A), we used expression data (Chantzoura et al., 2015) to select genes with robust expression in MEFs or at least 4-fold upregulated expression in reprogramming cells. The custom siRNA library comprised siRNAs targeting 300 chromatin-associated factors. For each target, three independent siRNAs were pooled for transfections (Table S1).

We were specifically interested in the early phase of reprogramming, as chromatin is known to confer epigenetic stability to somatic cells. To test the function of chromatin-associated genes in early reprogramming, we used a relatively fast reprogramming system (Esteban et al., 2010, Vidal et al., 2014) in which colonies can be detected after 6 days of reprogramming (Figures 1B and S1A). Day 6 colonies show round, symmetric morphologies and robust expression of early markers CDH1, SSEA1, and SALL4, with gene expression of *Nanog* and *Esrrb* appearing later (Figures S1B–S1D). The specific staining of CDH1 and SALL4, respectively, at the cell surface and in the nucleus, strongly increased between days 3 and 6 (Figures 1B and S1), representing a suitable readout for the early phase of reprogramming.

The expression of genes was knocked down using siRNAs in MEFs infected with an inducible OSKM cassette lentivirus. Reprogramming was induced with doxycycline (dox) for 6 days (Figure S1A). The siRNA library consisted of six 96-well plates, with each plate containing seven non-targeting (nt) siRNA negative controls and three positive controls (siRNAs targeting *Trp53*, *Oct4*, and *c-Myc*). The screen was performed in quadruplicate. After 6 days of reprogramming, samples were fixed, stained for CDH1 and SALL4, and imaged using an automated high-content microscope. This allowed quantitation of morphology features such as colony size, symmetry and shape, and marker intensities, but also texture features. After data processing and colony feature extraction, the data were *Z* score normalized per plate (Bakal et al., 2007) and subjected to further analysis (Figure 1A).

To test the system, we disrupted reprogramming by knocking down OSKM factors *Oct4* (si*Oct4*) and *c-Myc* (si*Myc*). We also knocked down *Trp53* (si*Trp53*), which is expected to enhance reprogramming (Marion et al., 2009). si*Oct4* and si*Myc* colonies are flat and irregularly shaped, and show less intense SALL4 and CDH1 staining compared with the control (Figure 1C). As expected, the number of colonies observed in si*Oct4* and si*Myc* in our *Z* score-ranked data was low (Figure 1D). The si*Trp53* control showed a variable but positive effect on the number of colonies. These data confirm that colony morphology and early marker expression can be used as a readout for a disruption in the early reprogramming network. The screening focuses on early reprogramming events, and therefore it is unlikely to capture factors required later to attain full pluripotency.

### High-Content Microscopy Reveals Five Major Phenotypes of Colony Formation

The high-content analysis allowed us to measure a number of colony features (Table S2). This constitutes a multi-dimensional phenotypic space for analysis across many conditions or perturbations (Boutros et al., 2015), and for the identification of functionally connected genes and processes (Mulder et al., 2012, Wang et al., 2012).

**Figure 2:**
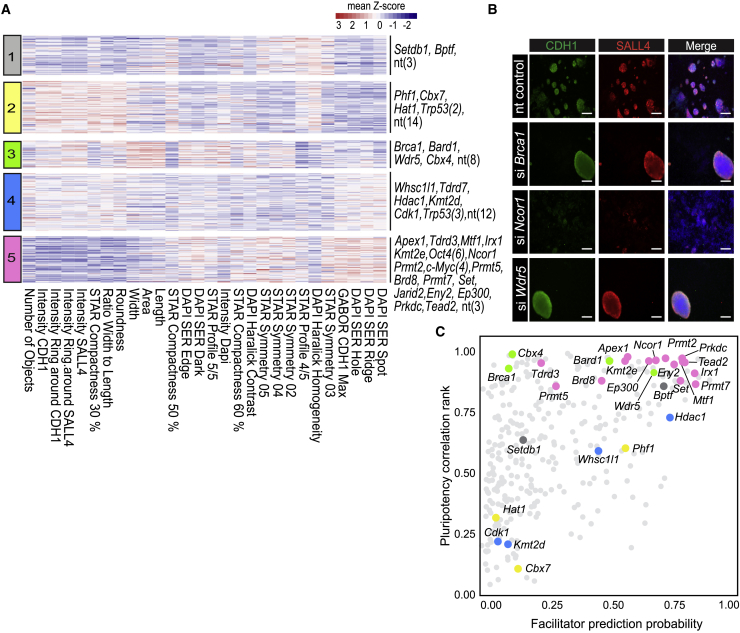
High-Content Microscopy Screen Reveals Five Major Phenotypes of Colony Formation (A) An average *Z* score for selected high-content features was calculated from quadruplicate samples (independent siRNA transfections from same experiment) and represented in a heatmap. Features are clustered by Euclidean distance and rows are clustered by K-means. Cluster number (left) and hits of the screen and the controls (right, number in brackets) are indicated. (B) Examples of knockdowns showing different phenotypes. Scale bars, 200 μm. (C) Pluripotency-associated hits were selected based on a combination of a probability prediction by machine learning based on known reprogramming facilitators (x), and a correlation analysis with the positive and negative controls (y). Selected top-hits are colored according to the cluster number (A; cf. Tables S3 and S4, Figure S2). Each data-point represents average of four replicates (A).

We first defined the set of most discriminating features based on feature-to-feature pairwise correlations (Supplemental Information; Table S3). Using hierarchical (Figure S2) and K-means clustering (Figure 2A) we observed five main clusters that display different levels of pluripotency markers, number of colonies, symmetry features (ratio width to length, roundness), symmetry, threshold compactness, axial and radial (STAR) morphology features, and textural features (SER, Haralick, Gabor). Some textural features capture the distribution of intensity patterns across the image. These phenotypes can be associated to structural changes in subcellular components (membranes, nucleoli, and chromatin). Other features or filters can detect how densely packed cells or cellular structures are (STAR features, see Supplemental Experimental Procedures). Although not all features have an intuitive biological interpretation, they are useful to discriminate cellular phenotypes and patterns not easily noted by the human eye in an unbiased manner (Boutros et al., 2015).

Cluster 1 knockdowns have few colonies, in addition to lower intensities for SALL4 and CDH1 compared with clusters 2–4, suggesting a potential defect in reprogramming. Cluster 1 also shows lower scores for nuclear texture features (DAPI SER), suggesting structural changes in nuclei. More than half of the nt controls are found in cluster 2, which shows a high number of small, round, and compact colonies, and a robust expression of CDH1 and SALL4 (Figures 2A and 2B). Cluster 3 displays fewer, large colonies (high width, area, length scores), impaired compactness (STAR compactness), and detectable SALL4 and CDH1 expression (Figure 2A, cf. *Brca1* and *Wdr5*, Figure 2B). For cluster 4, no dramatic changes are observed compared with cluster 2, except for some DAPI SER texture features that score lower in cluster 2. Clusters 2 and 4 can be considered normal reprogramming, since they include most nt controls. However, they could still contain some enhancer factors, since si*Trp53* also resides there. Cluster 5 is characterized by substantially lower SALL4 and CDH1 staining intensities and a low number of irregularly shaped, less round (ratio with-to-length), and less compact colonies (cf. *Ncor1*, Figure 2B). Phenotypes of cluster 5 suggest a severe reprogramming impairment, as confirmed by the presence of si*Myc* and si*Oct4* controls in that cluster.

Positive controls present in our screening (*Trp53*, *Myc*, and *Oct4*) are key for reprogramming. We reasoned that knockdown (high content) phenotypes similar to those of controls could potentially indicate a role in reprogramming progression. Therefore, we individually correlated each of the knockdowns to the positive controls (*Trp53*, *Myc*, and *Oct4*) based on all high-content features shown in Figure 2A using Pearson correlation (Table S4). Correlation scores of each knockdown to the positive controls were combined into a single ranking score for each knockdown (Figure 2C, y axis; cf. Experimental Procedures, Table S4). From this approach, we selected the 20 top-ranking knockdowns (Figure 2C).

In addition, the high-content phenotypes associated with known reprogramming facilitators (Table S4) present in our library (e.g., *Tet2*, *Jarid2*, and *Setdb1*), were used to train two independent machine-learning algorithms. Based on the high-content phenotypes of these facilitators, the algorithms classify the rest of the knockdowns, assigning them a score. Such a score indicates how well each knockdown is predicted to facilitate reprogramming (Figure 2C, x axis; cf. Supplemental Information; Table S4). We selected candidates from among the top-score predictions, but also some lower ranking siRNAs, because they may represent roadblocks rather than facilitators of reprogramming (Figure 2C and Table S4). In total, 30 siRNAs were selected for an orthogonal transcriptome screen.

### A Transcriptome-Based Secondary Screening Uncovers Highly Correlated Phenotypes

**Figure 3:**
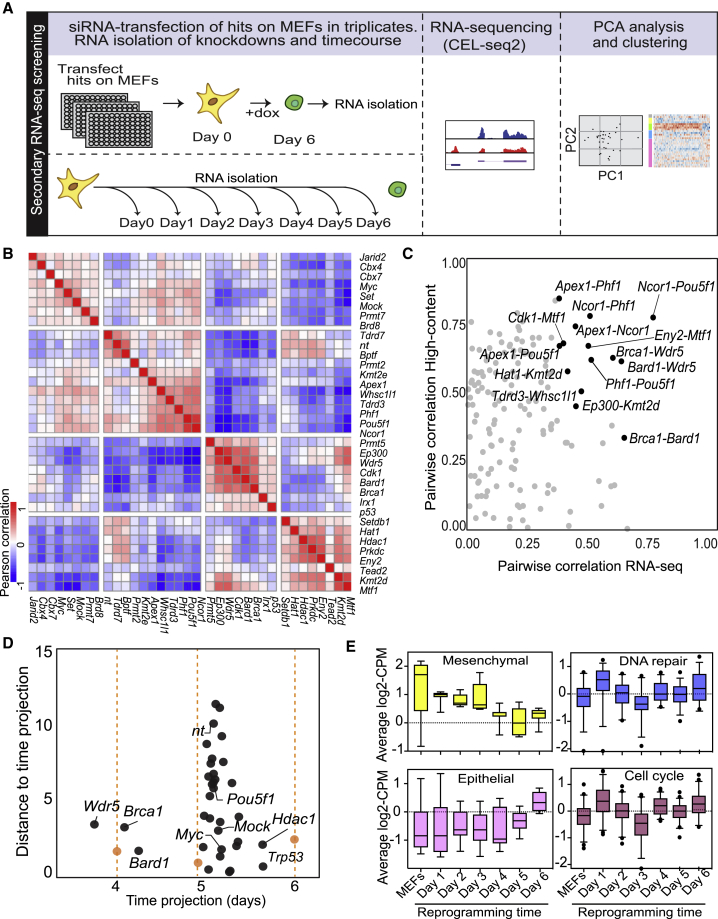
Transcriptome-Based Secondary Screening and Time Course Expression Analysis (A) Overview of experimental design. Selected hits (30) and controls were transfected in triplicate and cultured until reprogramming day 6. The transcriptomes were analyzed together with a time series of control cells. (B) siRNA-to-siRNA Pearson correlation heatmaps based on transcriptomes. (C) Scatterplot representing pairwise siRNA correlations of gene expression values (x axis) and high-content imaging features (y axis). siRNA pairs with highest correlations in both approaches are highlighted. (D) Progression of reprogramming in knockdown cells (black) compared with cells of the time course (orange), based on PCA analysis of the transcriptomes and the projection of all data points on a curve fitted to the time course. (E) Boxplots representing log transformed and normalized gene expression values from the CELSeq2 time course dataset show expression of different groups of genes (Experimental Procedures, Table S5, Figures S3 and S4). The box represents the interquartile range and the line is the median. (B)–(E) Averages of three independent RNA sequencing replicates.

We hypothesized that the phenotypes observed by microscopy might be reflected in their transcriptomes. Cells were transfected with siRNAs in triplicate, and day 6 RNA samples were subjected to CEL-Seq2-based RNA sequencing (Hashimshony et al., 2016). In addition to the 30 knockdowns, we also sequenced a day-by-day reprogramming time course of control cells (Figure 3A).

We performed principal-component analysis (PCA) to visualize transcriptome similarity based on the top 200 most variable transcripts across all samples (Figure S4A). Using these most variable transcripts, knockdown samples were clustered based on pairwise Pearson correlations (knockdown-to-knockdown; Figure 3B). This revealed groups of knockdowns with highly correlated transcriptomes, as noted in the intense red squares (Figure 3B). To analyze how the high-content phenotypes relate to the transcriptomes, we also calculated pairwise correlations of the same 30 siRNAs based on all high-content features (Figure 3C). This identified knockdown pairs that correlated in both their colony phenotype and their transcriptome (Figure 3C, highlighted in black). The strongest correlations were observed between the *Ncor1*-*Oct4* pair and a triplet consisting of *Wdr5*, *Brca1*, and *Bard1*. siRNAs with similar phenotypes (transcriptome and colony morphology) may reveal functional interactions. Indeed, NCoR1 has been shown to interact physically with OCT4 and MYC (Zhuang et al., 2018). However, the functional relationships between *Brca1*, *Bard1*, and *Wdr5* were unknown, therefore we decided to follow up on the effects of these siRNAs.

We performed siRNA deconvolution experiments measuring the number of SALL4-positive colonies of three independent siRNAs for *Wdr5*, *Brca1*, and *Bard1* to exclude off-target effects. This analysis resulted in phenotypes similar to the pooled siRNAs in at least two out of three siRNA sequences with the same target (Figure S3). In addition, high knockdown efficiencies of the *Brca1*, *Bard1,* and *Wdr5* mRNA targets were verified at day 3 of reprogramming (Figure S3). As reprogramming is a dynamic process, we wondered how cells progress toward the iPSC state in each of the knockdown conditions. Notably, in the PCA analysis, principal component 2 (PC2) correlates strongly with time (r^2^ = 0.81; Figures S4A and S4B). To model the reprogramming progression in each knockdown, we fitted a polynomial function to the time course data, and projected all other data on this fitted time line by shortest distance (Figures 3D and S4C; Experimental Procedures). The distance to the time line reflects transcriptome changes that are unrelated to progression of reprogramming. Such effects are to be expected as the siRNA library consisted of epigenetic and transcriptional regulators that may also affect processes not directly related to reprogramming. Most siRNA knockdowns, including the nt and mock transfected controls, have a transcriptome that is similar to control cells between day 5 and 6 of reprogramming, indicating a mild non-specific effect of transfection. Silencing *p53* and *Hdac1* modestly speeds up reprogramming relative to nt controls (Figure 3D). *Wdr5*, *Brca1*, and *Bard1*, show a strong delay in reprogramming with a short distance to the time projection of control cells, suggesting that most expression differences in these siRNA cells are due to the delay in reprogramming. si*Wdr5* cells were comparable with normal cells between day 3 and 4, while si*Bard1* and si*Brca1* cells were between day 4 and 5 (Figure 3D).

Next, we analyzed the gene expression changes associated with the colony morphologies of our high-content screen. For this, we identified the differentially expressed genes for each of the five morphological clusters (Figure 2A) using the secondary screen mRNA expression data. This highlighted a strong deregulated transcription program specific for morphological cluster 3 (containing *Wdr5*, *Brca1*, and *Bard1*) that was enriched for genes involved in cell-cell adhesion (Figure S4D; Table S5). Besides cell adhesion genes, this transcription program also contained *Zeb1* and *Twist2*, two regulators of mesenchymal cell fate. Indeed, genes in cluster 3 showed deregulation of mesenchymal and epithelial gene expression compared with the other clusters (Figure S4E; Table S5).

We then analyzed our time series data to relate the early block observed with si*Wdr5*, si*Brca1*, and si*Bard1* to the dynamics of genes involved in the MET, DNA repair, and cell-cycle regulation (Table S5). The block of reprogramming is observed around day 4 (Figure 3D), coinciding with the time of a major decrease of mesenchymal gene expression and preceding the activation of epithelial markers (Figure 3E). For DNA repair and cell-cycle genes there is an early wave of increased expression followed by downregulation, whereas a set of randomly selected genes are stably expressed over the time course of reprogramming (Figure S4F). This raised the possibility that WDR5, BRCA1, and BARD1 affect the repression of mesenchymal gene expression during early reprogramming. Moreover, based on the phenotypic and molecular co-correlation data we hypothesized that *Wdr5*, *Brca1*, and *Bard1* cooperate to control early stages of reprogramming.

### BRCA1, BARD1, and WDR5 Functionally Interact during Early Reprogramming

**Figure 4:**
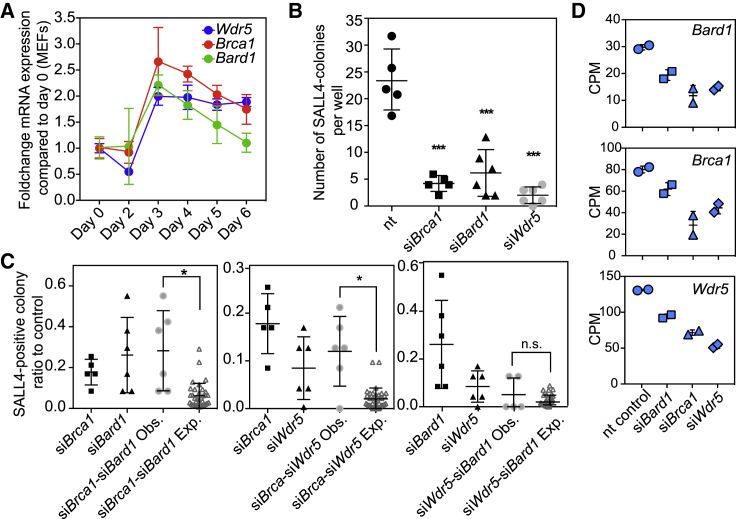
BRCA1, BARD1, and WDR5 Functionally Interact in Early Reprogramming (A) Gene expression of *Brca1*, *Bard1*, and *Wdr5* measured by qRT-PCR. Fold change was calculated relative to MEFs (day 0) gene expression. Each data point represents the mean value ± SD of a duplicate from the same experiment. (B) Dot plot showing the number of SALL4-positive colonies measured by in-cell western in control and *Brca1*, *Bard1*, and *Wdr5* knockdowns at day 6. Replicates are independent transfections from the same experiment. Statistical significance determined by one-way ANOVA (^∗∗∗^p < 0.0005). (C) SALL4-colony ratios of the single and double knockdowns compared with the non-targeting (nt) control, measured by in-cell western at day 6. Functional interaction is determined by comparing the mean difference in double knockdown colony ratios: observed (Obs.) versus expected (Exp.). Replicates are independent transfections of an experiment performed at least twice. Statistical significance (^∗^p < 0.05) was calculated with two-tailed t-test. (D) Dot plots to show *Wdr5*, *Brca1*, or *Bard1* gene expression as counts per million (CPM) reads in si*Bard1*, si*Brca1*, si*Wdr5*, and nt control. (B)–(D) Each dot or data point represents a replicate and the lines represent mean ± SD.

We asked whether *Wdr5*, *Bard1*, and *Brca1* genes have similar expression dynamics during early reprogramming. Interestingly, the three genes follow a similar qRT-PCR profile, peaking in expression at day 3, and then slowly going down (Figure 4A). To test the possibility of a functional interaction between these genes, the effect of double knockdowns was measured and compared with the effect of the single knockdowns regarding the number of colonies formed. In these experiments, the total amount of siRNA was kept the same (Experimental Procedures), preventing siRNA overloading and making sure that transfection conditions were comparable. All three single knockdowns displayed a significant reduction in number of SALL4-positive colonies compared with the nt control (Figure 4B). *Brca1-Bard1* double knockdown showed significantly more colonies than expected if the siRNAs were to have independent effects on the relative number of colonies (Figure 4C, left). This result was anticipated, as BRCA1-BARD1 are well-known physical interactors (Wu et al., 1996). Similarly, for both the *Wdr5-Brca1* and the *Wdr5-Bard1* double knockdowns, we also observed more colonies than expected, and this result was statistically significant for *Wdr5-Brca1* (Figure 4C). These results implicate the three genes in the same functional pathway. To test whether WDR5 is directly activating *Brca1* and *Bard1* gene expression, we determined the *Brca1* and *Bard1* expression levels after *Wdr5* knockdown (Figure 4D). Indeed, we found that this is the case at day 3, but also found that, in response to either *Bard1* or *Brca1* depletion, *Wdr5* expression was decreased. Taken together, *Brca1*, *Bard1*, and *Wdr5* are co-expressed, mutually depend on each other, and interact functionally in reprogramming.

### WDR5, BARD1, and BRCA1 Are Functionally Connected in the DDR Pathway

**Figure 5:**
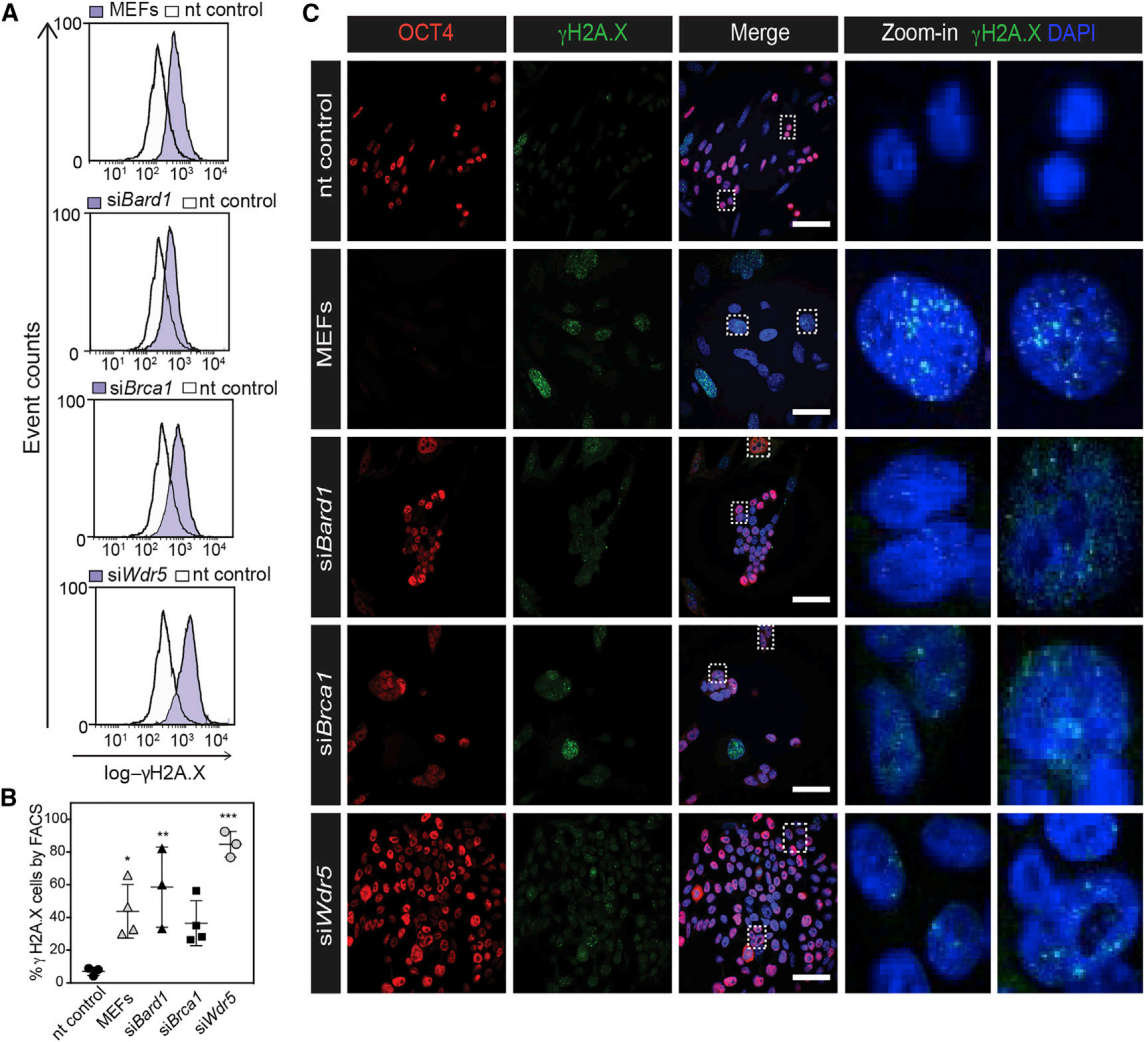
WDR5, BARD1, and BRCA1 Are Functionally Connected in the DNA Damage Response Pathway (A) Representative FACS histograms showing the cell distribution with log-intensity of γH2A.X in reprogramming populations measured in different conditions (white, nt; purple, siRNA). Each sample was measured in at least three independent experiments. (B) Dot plot representing the quantification of γH2AX-positive cells in each condition. Data points correspond to biological replicates from independent experiments. Statistical significance was determined by one-way ANOVA (^∗^p < 0.05, ^∗∗^p < 0.005, ^∗∗∗^p < 0.0005). (C) Confocal images of reprogramming cells at day 3, stained for γH2A.X (green) OCT4 (red), and DNA (DAPI, blue). Scale bars, 100 μm (left-middle). Zoom-in (right): magnification from inset, showing characteristic γH2A.X foci in all samples except nt control.

BRCA1 and BARD1 have a known function in double-strand break DNA repair. If BRCA1 and BARD1 functionally interact with WDR5, the prediction is that that all three knockdowns show an increase in DNA damage. The phosphorylated form of the histone variant H2A.X (γH2A.X) represents a reliable biomarker for DNA damage as it is an immediate response to the presence of double-strand breaks (Sharma et al., 2012). Therefore, we employed fluorescence-activated cell sorting (FACS) analysis to measure γH2A.X after knockdown (Figures 5A and 5B). Reprogramming cells (nt control) showed a significant decrease in DDR compared with non-reprogramming MEFs, in agreement with literature showing that reprogramming resolves DNA damage in somatic cells (Ocampo et al., 2016). Importantly, *Wdr5* knockdown showed a significantly increased level of γH2A.X compared with the control (Figure 5A). Nearly 90% of the cells harbor γH2A.X in *Wdr5*-depleted cells (Figure 5B, bottom panel). As expected, si*Brca1* and si*Bard1* also showed a high percentage of γH2A.X-positive cells (Figures 5A and 5B). We also visualized γH2A.X by immunofluorescence. At day 3 of reprogramming, knockdown cells and controls were stained for OCT4 and γH2A.X (Figure 5C). In agreement with the results from the FACS analysis, nt control transfected (OCT4-positive) reprogramming cells showed strongly reduced focal γH2A.X staining compared with the MEFs. In contrast, depletion of *Brca1*, *Bard1*, or *Wdr5* resulted in OCT4-positive, γH2A.X-positive cells (Figure 5C). These results confirm the association of *Wdr5*, *Brca1*, and *Bard1* with the DDR during reprogramming.

### WDR5, BRCA1, and BARD1 Affect MET and DNA Repair Gene Expression

**Figure 6:**
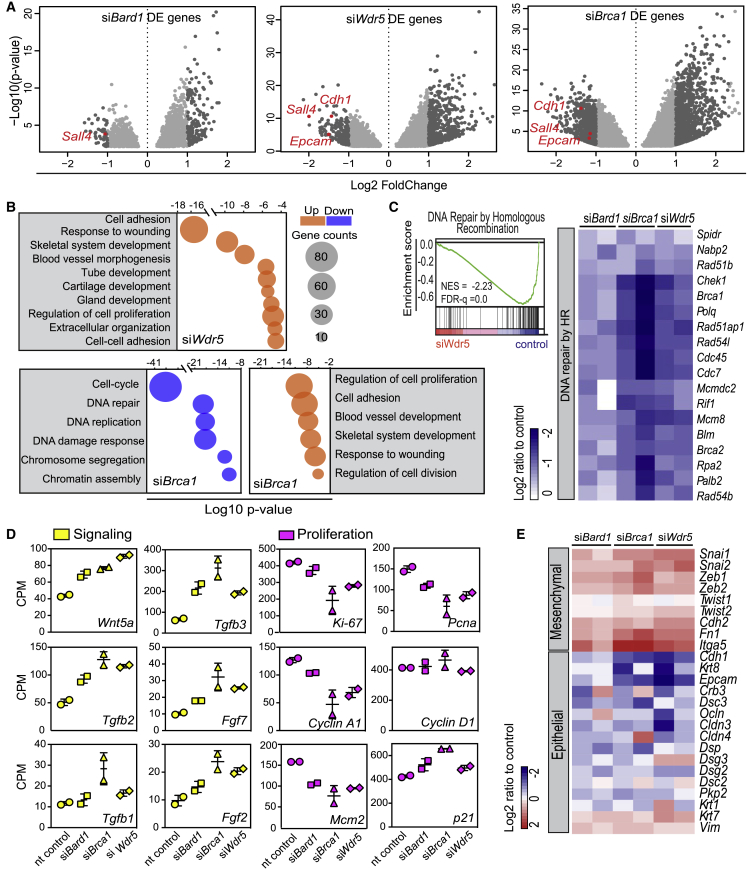
*Wdr5*, *Brca1*, and *Bard1* Depletion Affects Expression Profiles of MET and DNA Repair Genes (A) Volcano plots derived from two independent RNA sequencing replicates for si*Bard1* (left), si*Brca1* (middle), and si*Wdr5* differential gene expression at reprogramming day 3. Highlight: differentially expressed genes (log2-fold change ≥1, adjusted p < 0.05). (B) Bubble plot with enriched gene ontology terms (upregulated genes, orange; downregulated genes, blue) at day 3. Bubble sizes represent the number of genes. (C) Gene set enrichment analysis for DNA repair by homologous recombination (HR) comparing si*Wdr5* versus control transcriptomes (left). NES, normalized enrichment score; FDR, false discovery rate. Heatmap for si*Brca1*, si*Bard1*, and si*Wdr5* samples showing genes for DNA repair by HR (log2-ratio relative to control). (D) Gene expression (RNA sequencing CPM) of signaling genes (magenta) and cell proliferation markers (yellow) in control, si*Brca1*, si*Bard1*, and si*Wdr5* cells (each data point represents individual RNA sequencing replicates from independent experiments; bars, average ± SD). (E) Heatmap representing the log2-ratio of mesenchymal and epithelial gene expression of the three knockdowns relative to control. See Figure S5 and Table S6.

To gain more insight into the *Wdr5*, *Brca1*, and *Bard1* phenotypes and their link to MET (Figures 3E and S4C), we performed deep RNA sequencing at day 3 and day 6 of reprogramming. We called differentially expressed genes and found 753, 1,555, and 205 genes deregulated in, respectively, *Wdr5*, *Brca1*, and *Bard1* knockdown cells following 3 days of OSKM induction (Figure 6A). *Wdr5*-, *Brca1*-, and *Bard1*-depleted cells showed reduced expression of early pluripotency genes such as *Sall4*, and epithelial genes *Cdh1* and *Epcam* (Figure 6A).

Differentially expressed genes in each knockdown were further probed for overrepresented gene ontology (GO) classes (Figure 6B; Table S6). *Brca1* knockdown causes a reduction in gene expression related to the cell cycle, response to DNA damage, and DNA repair (Figure 6B). We asked whether the effects on the DDR (Figure 5) are reflected in the transcriptome of si*Wdr5* as well. To test this, DNA repair genes were probed in a gene set enrichment analysis (Mootha et al., 2003, Subramanian et al., 2005) comparing si*Wdr5* and control transcriptomes. Indeed, the negative normalized enrichment score indicated decreased expression of DNA repair genes in the si*Wdr5* compared with the control (Figure 6C, left). Furthermore, decreased expression of DNA repair genes in si*Wdr5* was similar to that of si*Brca1* and si*Bard*1, two well-known regulators of DDR (Figure 6C, right and Figure S5).

*Wdr5* and *Brca1* knockdowns shared a number of upregulated terms, including cell adhesion and developmental processes (e.g., skeleton or blood vessel development; Figure 6B). Regulation of cell proliferation was also affected in *Brca1*, *Bard1*, and *Wdr5* knockdowns. This GO term was enriched due to increased expression of, among others, *Tgfβ*-, *Wnt*-, *Bmp*-, and *Fgf*-encoded growth factors (Table S6). In the RNA sequencing data from the knockdowns, we indeed observed higher expression values for these signaling factor genes compared with controls (Table S6; Figure 6D, left). These growth factors decrease cell proliferation and are involved in epithelial-to-mesenchymal transitions (EMT) (Barrallo-Gimeno and Nieto, 2005), potentially counteracting the MET required for reprogramming. Several cell proliferation markers, including *Pcna*, *Ki-67*, and *Mcm2* showed decreased expression in all three knockdowns, while *p21* (*Cdkn1a*) was upregulated (Figure 6D, right). These data suggest an impaired MET upon *Brca1*, *Bard1*, and *Wdr5* depletion.

We assessed the gene expression levels of mesenchymal and epithelial markers in the three knockdowns and observed a clear increase in mesenchymal gene expression in the *Wdr5*, *Brca1*, and *Bard1* knockdown cells relative to control cells (Figures 6E and S5). Some epithelial genes were decreased (*Cdh1*, *Epcam*, and *Krt8*), whereas others did not change substantially or were increased (Figures 6E and S5). Together, our data indicate that WDR5, BRCA1, and BARD1 cooperate in DDRs and that their absence affects MET progression during an early phase of reprogramming.

## Discussion

Previously it has been shown that WDR5, a core component of SET-MLL methyltransferase complexes, interacts with OCT4 to activate the pluripotency network through H3K4 me2/me3 deposition (Ang et al., 2011). Our study shows that WDR5 also functionally interacts with BRCA1-BARD1 to control DDR, and that MET is severely perturbed in the absence of these factors. The nature of the interaction remains to be elucidated. One possibility is that *Brca1* and *Bard1* are direct or indirect targets of the SET/MLL complexes, of which WDR5 is a subunit. In line with this possibility, chromatin immunoprecipitation analysis showed that WDR5 binds regulatory regions of *Brca1*, *Bard1*, and other genes involved in DNA repair (Ang et al., 2011). Moreover, we show that *Brca1* and *Bard1* transcripts are downregulated after silencing *Wdr5*. In addition, BRCA1 and BARD1 are involved in mitotic spindle organization and checkpoint gene regulation (Jin et al., 2009, Joukov et al., 2006, Wang et al., 2004), and MLL/WDR5 has been implicated in cell-cycle regulation, mitotic progression, and proper chromosome segregation as well (Ali et al., 2014, Ali et al., 2017, Liu et al., 2010).

DNA damage in reprogramming is, at least partly, associated with senescence (Ocampo et al., 2016), which can be rapidly induced by oxidative stress in primary cells (Xu et al., 2016). In agreement, low oxidizing conditions reduce the reprogramming barrier imposed by senescence (Utikal et al., 2009). BRCA1-BARD1 and WDR5 might alleviate a senescence-related block of reprogramming. Likewise, the requirement of a DDR could be related to the faster proliferation rates acquired early on in reprogramming (Polo et al., 2012, Ruiz et al., 2011). Embryonic stem cells, which proliferate in a similar fashion, indeed require additional genome surveillance mechanisms to cope with fast DNA replication (Ahuja et al., 2016).

Our study adds to the notion that colony morphology is linked to pluripotency (Abagnale et al., 2017, Kato et al., 2016, Narva et al., 2017) and is regulated by adhesion molecules, extracellular matrix, and cytoskeleton forces. Upon differentiation, these processes orchestrate morphological changes such as loss of colony compaction, increase of cell area, and colony flattening, together with changes in the pluripotency network (Narva et al., 2017). Therefore, colony morphology is an important readout for reprogramming quality. Moreover, screening of such multi-dimensional phenotypes is a powerful approach to identify functional interactions between genes. *Brca1*-, *Bard1*-, and *Wdr5*-depleted cells gave rise to fewer yet bigger, flat, symmetric colonies. One possibility is that these morphological changes are associated with a failure to downregulate mesenchymal cell adhesion molecules. In addition, these cells fail to activate epithelial genes. Our study suggests a link between DDR and MET through *Brca1-Bard1* and *Wdr5* early in reprogramming. However, we cannot rule out that the MET phenotype is an indirect consequence of DDR impairment. Interestingly, the converse process of EMT may relate to DNA damage in kidney disease (Slaats et al., 2014) and cancer cells in culture (Chiba et al., 2012). Future work will further explore these relationships as well as gene-gene interactions that modify the phenotypic plasticity of reprogramming to induced pluripotency.

## Experimental Procedures

### Data and Software Availability

All sequencing data are available at the GEO repository Superseries number GSE118680.

The code to reproduce reprogramming facilitator predictions by machine learning is available at https://github.com/simonvh/facilitators-penalosa-ruiz/. The code to reproduce the timeline projection is available at https://github.com/TimEVeenstra/Time-Curve-Projection/(https://doi.org/10.5281/zenodo.1405746).

### MEF Reprogramming and Culture Media

Passage 1–2 MEFs were seeded at a density of 10,000 cells per cm^2^. Next day, MEFs were transduced at an MOI of 1 with Tet-STEMCCA lentivirus (Sommer et al., 2009), rtTA (Addgene, no. 20342), and 8 μg mL^−1^ polybrene. Next day (day 0), cells were transferred to either 1% gelatin-coated plates or mitotically inactive feeder cells, in reprogramming medium (Vidal et al., 2014).

### siRNA Transfections and siRNA Screenings

A custom Silencer siRNA library targeting around 300 mouse genes encoding chromatin factors was designed (Thermo Scientific/Ambion, Table S1) and distributed in six plates. Each gene in the library was targeted with three different siRNAs, which were pooled for transfection. For the high-content screening, the six pooled plates were transfected in quadruplicate. Every plate contained the following controls: si*Oct4* (si*Pou5f1*), si*Myc*, si*Trp53*, and seven nt controls. Reverse transfections in a 96-well plate format were performed as follows: 20 μL of transfection mix was prepared in each well before adding the cell suspension. This transfection mix consisted of 40 nM of pooled siRNAs (considering 120 μL final volume), and 0.26 μL RNAiMAX lipofectamine (Thermo Scientific) diluted in Optimem (Thermo Scientific). After incubation for 10 min, 100 μL of cell suspension (3,000–6,000 cells) were added to each well. For transfections in a six-well plate format, the protocol was scaled up accordingly. Before adding 1.8 mL cell suspension with 100,000 cells, 220 μL transfection mix was incubated in the wells for 10 min. The transfection mix consisted of 4 μL RNAiMAX and a final concentration of 40 nM siRNA, all diluted in Optimem.

### Immunostaining

Cells were cultured in 96-well Cell Carrier plates for microscopy (PerkinElmer). After 6 days of reprogramming, cells were washed with PBS and fixed with 4% paraformaldehyde (PFA) for 15 min. After blocking and permeabilization, samples were incubated overnight with mouse anti-CDH1 (Cell Signaling, 14472) and then with goat anti-mouse Alexa 488 for 2 h. Staining with rabbit anti-SALL4 (Abcam, ab29112) was done overnight, followed by 3 h incubation with goat anti-rabbit Alexa 568 and 40 μg mL^−1^ DAPI. After antibody incubations, the cells were washed twice with PBS.

### High-Content Image Acquisition and Feature Selection

Plates were imaged with an Opera High-content Screening System (PerkinElmer) with a 4× air lens. Images were imported into the Columbus software platform (PerkinElmer). To segment colonies imaged on multiple z planes, we used the maximum projection of z planes. SALL4 staining was used to find and segment the colonies. Automated image analysis was used for image region segmentation and for extraction of shape and morphology features. Image regions touching the edge were removed. For more details, see Supplemental Information. After extracting all features for every plate from the automated pipeline, a *Z* score normalization was applied per plate (Bakal et al., 2007) based on the mean values per feature. To select relevant features, a feature-to-feature Pearson correlation was calculated. Features with a high pairwise correlation (>0.8) were considered redundant.

### RNA Sequencing and Analysis

CEL-seq2 sample preparation (Hashimshony et al., 2016) was performed with a few adaptations (see Supplemental Information). Transcripts were mapped to *Mus musculus* genome version mm10 with Bowtie2 (Langmead and Salzberg, 2012), UMI corrected using standard settings of the CELseq2 pipeline (https://github.com/yanailab/CEL-Seq-pipeline), and matched to the gencode.vM13.annotation transcriptome. To relate knockdown data points to the progression of reprogramming, the transcriptomes were subjected to PCA. PC1 and PC2 were swapped (x axis: PC2) and all data (knockdown and time series) were rotated 15° (cf. Figure S4B). A second-order polynomial curve was fitted to the time series (days 2–7), and all data points were projected on this curve (script: https://doi.org/10.5281/zenodo.1405747). For each data point, the projected x coordinate was used as a proxy for time, whereas the distance to the fitted time line (calculated using Pythagoras' theorem) was used as a proxy for gene expression differences unrelated to the process of reprogramming.

### FACS Analysis of DNA Damage

Reprogramming MEFs were transfected with siRNAs in six-well plates. After 3 days, cells were fixed on ice with 1% PFA for 15 min and incubated with 70% ice-cold ethanol at −20°C for 2 h. Samples were then incubated with 100 μL mouse anti-phospho-H2AX (Millipore, diluted 1:100 in 0.25% BSA 0.3% Triton/PBS) overnight at 4°C. Subsequently, cells were washed and stained with 100 μL Alexa 488 goat anti-rabbit 488 (diluted 1:500) for 2 h at room temperature. Finally, samples were incubated with propidium iodide overnight in a fridge and sorted using an FC 500 (Beckman Coulter) machine. Data analysis was done with Flowing software v.2.5. As positive control, reprogramming MEFs were treated with 400 μg mL^−1^ mitomycin C for 3 days.

### Double Knockdowns and Functional Interactions

Reprogramming was started in 48-well plate formats, with transfection reagents and number of cells scaled accordingly. For the double knockdown, a mixture of two targeting siRNAs was used in a final concentration of 40 nM, meaning 20 nM of each siRNA. The corresponding single knockdowns were performed with 20 nM siRNA target + 20 nM nt control siRNA, to make it equivalent to the individual siRNA dose in the double knockdowns. In this way, individual as well as final siRNA concentrations are comparable in double and single knockdowns (Mulder et al., 2012). The observed SALL4-colony ratio was calculated dividing the double knockdown number of colonies by the average number of colonies of the control (six biological replicates). The expected SALL4-colony ratio was calculated by multiplying the ratios of the single knockdowns (Mani et al., 2008). A p value of <0.05 (two-tailed t-test) was considered significant. See Supplemental Information for details.

## Author Contributions

Conceptualization, G.J.C.V., K.W.M., and G.P.-R.; Methodology, G.P.-R., V.B., G.J.C.V., K.W.M., C.B., and J.C.R.S.; Experiments, G.P.-R., V.B., J.P.G., S.W., and J.V.v.d.V.; Analysis, G.P.-R., V.B., J.P.G., G.J.C.V., S.J.v.H., and T.E.V.; Writing, G.P.-R., G.J.C.V., and K.W.M., with input from all authors.

## Supplemental Information

Document S1. Supplemental Experimental Procedures, Figures S1–S5, and Tables S1–S6

Table S1. siRNA Library, Related to Figures 1 and 2 Whole siRNA Silencer library arrays as obtained from Thermo Scientific.

Table S2. Screening Complete Dataset, Related to Figure 2 High-content screening whole dataset before feature selection.

Table S3. Screening Selected Features, Related to Figure 2 High-content screening dataset after filtering features and *Z* score normalization and quadruplicate average.

Table S4. Hit Selection, Related to Figures 2 and 3 Contains different sheets with various steps to reach the hit selection from high-content by machine learning and correlation analysis.

Table S5. Secondary RNA sequencing_Screening_and_Time course, Related to Figure 3 Spreadsheet with whole RNA sequencing screening dataset with time course. It also contains part of the analysis derived from these data, including gene ontology and gene sets depicted in all boxplots in the manuscript.

Table S6. Bulk RNA Sequencing Analysis, Related to Figure 6 Upregulated and downregulated genes in siBrca1, siBard1, and siWdr5 detected by differential gene expression analysis on bulk RNA sequencing data at Day3. It also contains the gene ontology classification (GO) associated to deregulated genes.

Document S2. Article plus Supplemental Information
