# Supplementary material for: WDR5, BRCA1, and BARD1 Co-regulate the DNA Damage Response and Modulate the Mesenchymal-to-Epithelial Transition during Early Reprogramming
Source: Stem Cell Reports. 2019 Mar 14;12(4):743–56. doi: 10.1016/j.stemcr.2019.02.006 (PMC6449870; doi:10.1016/j.stemcr.2019.02.006)
Supplement: Document S1. Supplemental Experimental Procedures, Figures S1–S5, and Tables S1–S6 [file mmc1.pdf]

Stem Cell Reports, Volume *12*

Supplemental Information

WDR5, BRCA1, and BARD1 Co-regulate the DNA Damage Response and Modulate the Mesenchymal-to-Epithelial Transition during Early Reprogramming

Georgina Peñalosa-Ruiz, Vicky Bousgouni, Jan P. Gerlach, Susan Waarlo, Joris V. van de Ven, Tim E. Veenstra, José C.R. Silva, Simon J. van Heeringen, Chris Bakal, Klaas W. Mulder, and Gert Jan C. Veenstra

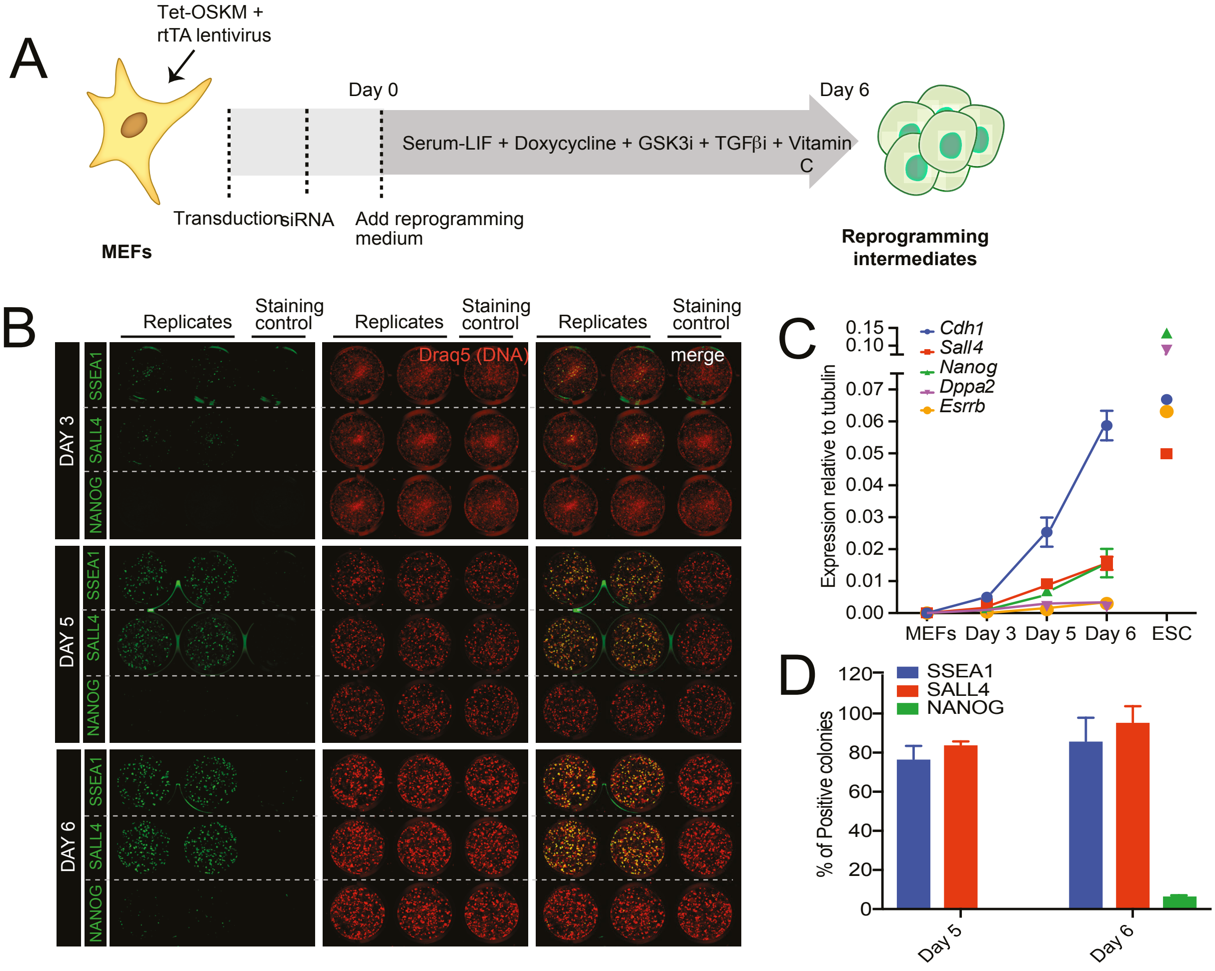

Figure S1. Characteristics of the reprogramming system. Related to Figure 1
(**A**) Schematic representation of the reprogramming protocol used. (**B**) In-cell western for reprogramming cells at day 3 (upper), Day 5 (middle) and Day 6 (lower) of the reprogramming protocol used in this study. For each timepoint samples were stained for SSEA1, SALL4 and NANOG in duplicate and counterstained with Draq5 (DNA). The staining control is the staining without primary antibody. (**C**) mRNA expression of early and later pluipotency markers of reprogramming timepoints. Expression levels are compared to those of Embryonic Stem Cells (ESC) at the right part of the plot. Data are presented as mean ± SD of biological replicates from same experiment (**D**) Quantification of positive colonies from B for each marker (SALL4, SSEA1 and NANOG) at Day 5 and Day 6. The calculation corresponds to the ratio of positive-colonies to Draq5. Day 3 was difficult to determine because of the fuzzy pattern. Data are presented as mean ± SD from replicates from the same experiment.

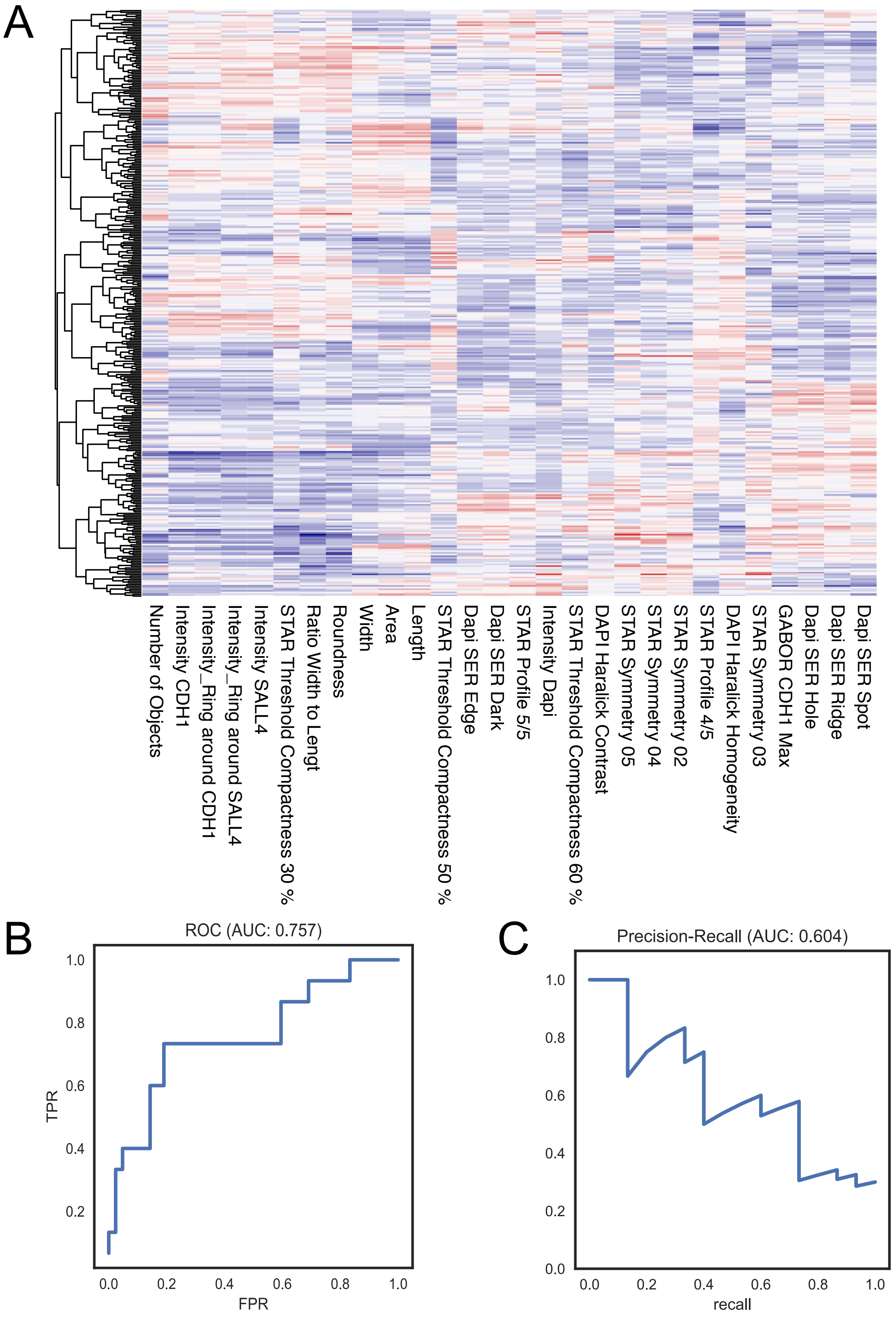

Figure S2. Unsupervised clustering and machine learning classification of high-content phenotypes. Related to Figure 2
(**A**) Hierarchical clustering based on Pearson correlation distance metrics applied to the whole screening dataset with filtered features.
The performance of the ensemble of the two machine learning classifiers in predicting reprogramming facilitators. Metrics were calculated based on the average probability in a leave-one-out cross-validation procedure. (**B**) The Receiver-Operator Curve (ROC), with an area under the curve (AUC) of 0.757. (**C**) The Precision-Recall curve with an AUC of 0.604.

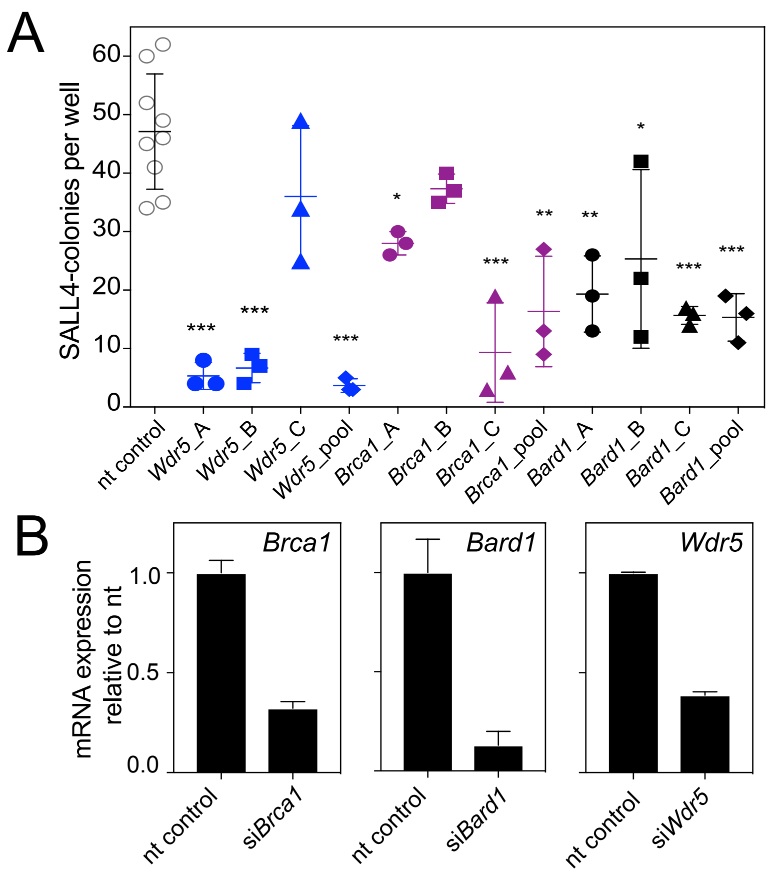

Figure S3. Analysis of *Wdr5*, *Brca1* and *Bard1* siRNA target specificities. Related to Figure 3.
(**A**) SALL4-positive colony formation assay for deconvolution of 3 siRNA sequences targeting *Wdr5* (blue), *Brca1*(magenta) or *Bard1*. In all cases, at least two out of the three sequences elicit the same colony-phenotype as the pooled siRNAs at day 6. Each dot represents one independent siRNA transfection from same experiment (**B**) Representative experiment showing knockdown showing efficiencies of si*Wdr5*, si*Brca1* or si*Bard1* for their targets analyzed at day 3 by RT-qPCR. The efficiency was calculated setting nt control mRNA levels to 1 and the siRNA percentage relative to that is plotted. Data represent mean ± SD from two independent transfections from the same experiment.

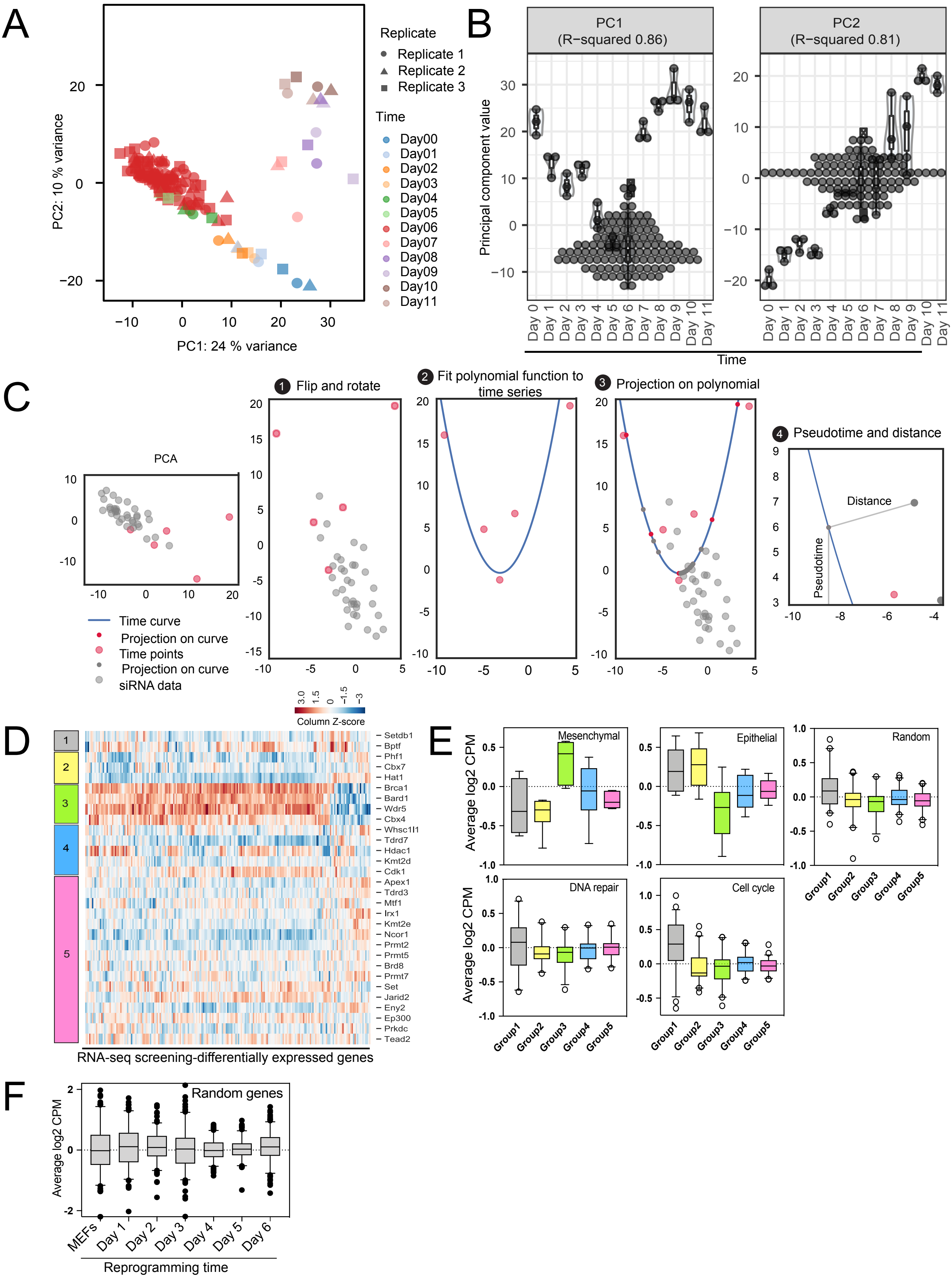
A
PC2: 10 % variance
PC1: 24 % variance
Replicate
Replicate 1
Replicate 2
Replicate 3
Time
Day00
Day01
Day02
Day03
Day04
Day05
Day06
Day07
Day08
Day09
Day10
Day11
B
PC1 (R−squared 0.86)
PC2 (R−squared 0.81)
Principal component value
Time
C
PCA
1 Flip and rotate
2 Fit polynomial function to time series
3 Projection on polynomial
4 Pseudotime and distance
Distance
Pseudotime
Time curve
Projection on curve
Time points
Projection on curve
siRNA data
D
Column Z-score
Setdb1
Bptf
Phf1
Cbx7
Hat1
Brca1
Bard1
Wdr5
Cbx4
Whsc1l1
Tdrd7
Hdac1
Kmt2d
Cdk1
Apex1
Tdrd3
Mtf1
Irx1
Kmt2e
Ncor1
Prmt2
Prmt5
Brd8
Prmt7
Set
Jarid2
Eny2
Ep300
Prkdc
Tead2
RNA-seq screening-differentially expressed genes
E
Average log2 CPM
Mesenchymal
Epithelial
Random
DNA repair
Cell cycle
Group1
Group2
Group3
Group4
Group5
F
Random genes
Average log2 CPM
MEFs
Day 1
Day 2
Day 3
Day 4
Day 5
Day 6
Reprogramming time

Figure S4. Secondary screening and time course RNAseq datasets analyses. Related to Figure 3.

(**A**) PCA of knockdowns (Day 6 red) together with time course showing all three replicates as different forms. (**B**) Correlation (r-squared value) with PC1 and PC2 dataset in A. PC1 and PC2 highly correlate with time. (**C**) Step-by-step time projection analysis from Figure 3D. The PCA plot (left-most panel, cf. Fig. S4A) is a two-dimensional graphical representation of gene expression differences among samples (data points). The gene expression differences observed with individual siRNAs may reflect gene expression differences related to the progression of reprogramming (for example a delay), whereas another component may be unrelated to the process of reprogramming. The analysis aims to separate these components. 1) First, the PCA plot was flipped and rotated 15 degrees to facilitate fitting a polynomial function. 2) A polynomial function (blue line, $y = 0.379\, x^2 + 2.90\, x + 3.42$) was fitted to the time course data points (red dots). 3) All data points, including all siRNA (gray) and time course (red) data points were projected into the polynomial function line (blue line), considering the shortest distance to it (perpendicular to the polynomial function). 4) The X coordinate represents pseudo-time (referred to as time projection in Fig. 3D), whereas the distance to the polynomial function is a representation of gene expression differences that are unrelated to the progression of reprogramming (**D**) Expression of differential genes across all siRNAs from the RNA-seq screening are presented in a heatmap. Differential genes are hierarchically clustered, and siRNAs are arranged according to the five high-content clusters in Fig.2A. The heatmap color code represents column Z-scores. The color code of clusters 1-5 corresponds to clusters in Fig. 2A. Each siRNA was averaged from three independent replicates prior to this analysis (**E**) Boxplots showing expression of some gene sets in clusters 1-5 from Fig.2A and S4-D. For each cluster, the expression values for all siRNAs in that cluster were pooled in a single boxplot. Color code corresponds to same clusters in S4D and Fig.2A. The gene sets were hypothesized from gene ontology classification (Table S5) derived from differential transcripts in D, and the fact that those processes change during early reprogramming. They are compared to a set of random genes (**F**) Boxplot from the average of three independent RNA-seq replicates in timecourse represent gene expression of 70 random genes compared to data from Fig. 3D-E. Unlike mesenchymal, epithelial, cell cycle and DNA repair genes (Fig.3E), the random genes do not show any dynamics in time. Median is the middle line and the boxes are the interquartile range.

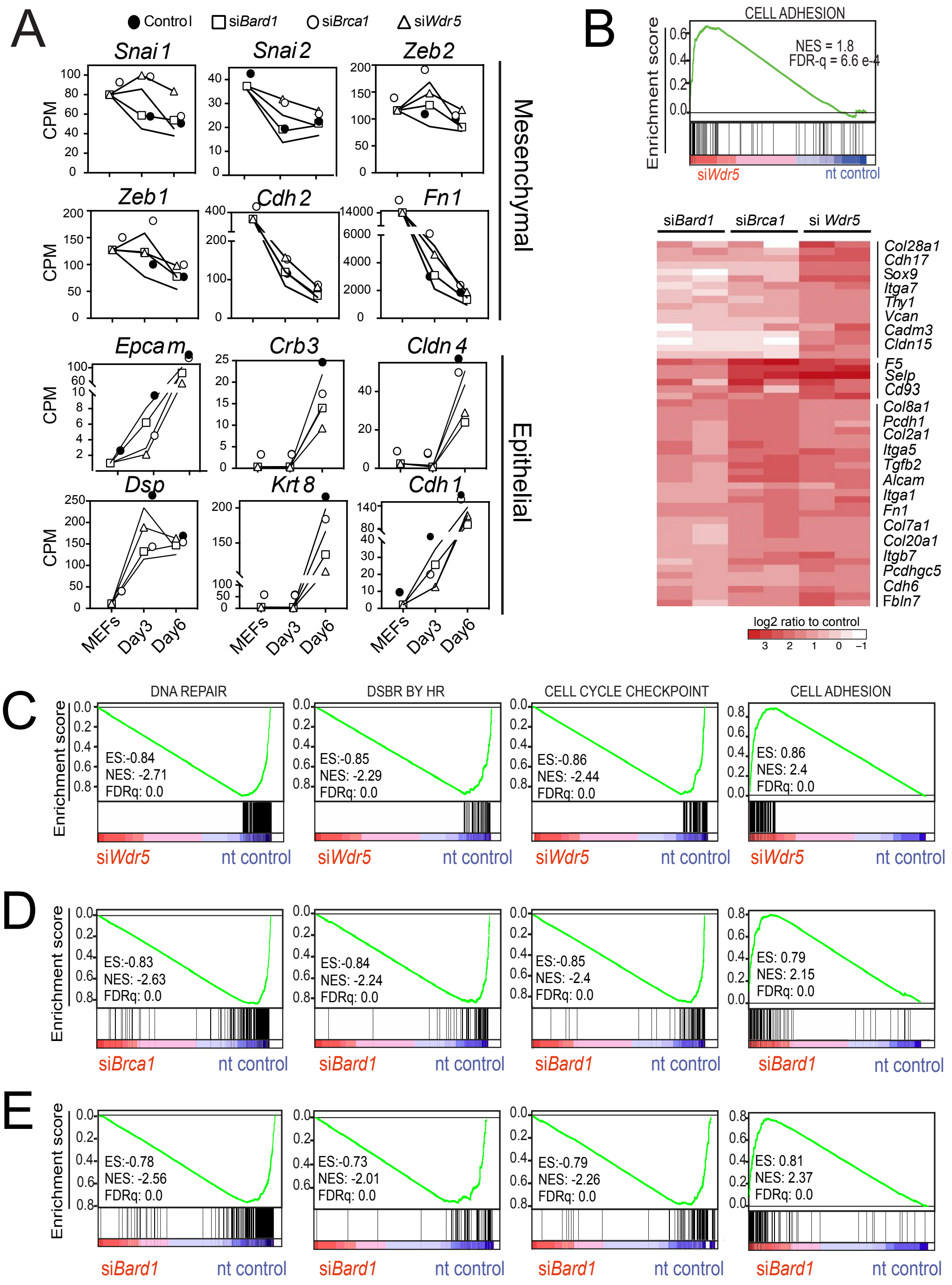

Figure S5. MET gene expression changes are related to cell adhesion molecules. Related to Figure 6

(**A**) Epithelial and mesenchymal gene expression in MEFs, Day 3 and Day 6 of reprogramming, comparing nt control, *Bard1*, *Brca1* and *Wdr5* depleted cells. CPM = counts per million reads. Data are presented as mean of two independent RNA-seq replicates (**B**) Leading edge analysis for *Wdr5* vs control, using the leading edge genes extracted from *Wdr5* vs control itself. Enrichment score for cell adhesion genes, comparing si*Wdr5* vs nt control (upper). Heatmap representing the log2-ratio compared to the control of cell adhesion genes in *Brca1*, *Bard1* and *Wdr5* depleted cells at day 3 of reprogramming (lower). (**C-E**) Leading edge analysis for *Wdr5, Brca1* or *Bard1* knock-downs vs control, using the core genes extracted from *Wdr5* vs control. The green curve represents the enrichment score. Enrichment Score (ES), Normalized Enrichment Score (NES), False Discovery Rate q value (FDRq).

SUPPLEMENTAL TABLES (all excel or text (.csv) files)

- **Table S1. siRNA library-Related to Fig. 1 and 2.** Whole siRNA Silencer library arrays as we obtained it from Thermo Scientific.
- **Table S2. Screening complete dataset. Related to Fig 2.** High-content screening whole dataset before feature selection.
- **Table S3. Screening selected features. Related to Fig. 2.** High-content screening dataset after filtering features and Z-score normalization and quadruplicate average.
- **Table S4. Hit selection. Related to Figs. 2 and 3.** Contains different sheets with various steps to reach the hit selection from high-content by machine learning and correlation analysis.
- **Table S5. Secondary RNAseq screening and_timecourse. Relates to Fig. 3.** Spreadsheet with whole RNA-seq screening dataset with time course. It also contains part of the analysis derived from these data, including gene ontology and gene sets depicted in all boxplots in the manuscript.
- **Table S6. Bulk-RNA-seq analysis. Relates to Fig. 6.** Upregulated and downregulated genes in si*Brca1*, si*Bard1* and si*Wdr5* detected by differential gene expression analysis on bulk RNA-seq data at Day3. It also contains the gene ontology classification (GO) associated to deregulated genes.

SUPPLEMENTAL EXPERIMENTAL PROCEDURES

MEF Reprogramming

MEF medium consisted of DMEM (High glucose, GlutaMAX, Thermo Scientific), 15% FBS, 1% Penicilin/Streptomycin, 1% non-essential amino acids (Thermo Scientific) and 100 nM ß-mercaptoethanol (Sigma).

In case of siRNA transfection, transduced cells were then incubated with the siRNA transfection mix for one more day before starting reprogramming. Reprogramming initiated by adding medium based on DMEM, 10% FBS (Hyclone), supplemented with 2µg·mL-1 doxycycline, 3 µM Chiron (GSK3-inhibitor), 0.25 µM Alk5i (TGF-ß inhibitor), 50 µg·mL-1 ascorbic acid and 1·103 U·mL-1 LIF. Considering this point as day 0, the protocol continued for 6 days for most of the experiments, unless specified otherwise.

High content feature analysis

Besides the geometric and basic morphology features, texture features for whole cell regions were calculated using the SER (Spot, Edges and Ridges) method. There are eight SER features; Spot, Hole, Edge, Ridge, Valley, Saddle, Bright and Dark. SER features measure occurrence of intensity patterns within the image. Another parameter used in the SER method is the scale, which is smoothing technique of filtered images. Texture features were normalized using the Kernel method, which means that the images are pixelwise divided by the smoothed original image.

Gabor filter features discriminate features based on differential intensities and it is similar to human eye pattern detection. Haralick features statistically describe similarities among neighbouring pixels (Pascual-Vargas et al., 2017)

STAR advanced morphological features (Symmetry properties, Threshold compactness, Axial properties, Radial properties) measure the symmetric distribution of the fluorescence intensities or textures and how compact these patterns are within the colonies. For the STAR profiles, the image region is subdivided in different sections and measured.

Intensity Ring features measured the intensity of SALL4 and CDH1 staining at the edges of the colonies as compared to the inner part of the colony.

For data visualization and clustering, the quadruplicate values per knockdown were averaged, including the controls. Hierarchical clustering and visualization was performed with pheatmap R package.

Hit selection

To select the top-hits we used a combined approach. One part of it was based on a top ranking score, which was calculated by correlation of knockdowns with positive controls (si*Oct4*, si*Myc*, siT*rp53*) and anti-correlation with non-targeting controls. Some of the top-ranking siRNAs were selected this way. The other part of the selection was based on a machine learning prediction. For that, we defined a set of known reprogramming facilitators based on literature (Table S4) and used these to train an ensemble of classifiers based on all features from Table S2. This ensemble consisted of an L2 penalized logistic regression and a random forest classifier. The hyperparameter of the L2 regularization of the logistic regression was set using three-fold cross-validation. The random forest model was trained with 1000 trees and a maximum of 10 features. The final probability was calculated as the mean of the predicted probability of the two classifiers. The performance was assessed by the ROC AUC (0.757) and the Precision-Recall AUC (0.604) based on leave-one-out cross-validation. This way, we selected the top-score siRNAs, but also some siRNAs scoring low, because these could potentially represent roadblocks.

Double knockdowns and deconvolution transfections

Reprogramming was started in 12-well or 48-well plate formats, with transfection reagents and number of cells scaled according to previous descriptions (see Experimental Procedures).

After six days of reprogramming, cells were fixed and stained (see in-cell western above). A CellProfiler script (Jones et al., 2008), kindly adapted by Jessie van Buggenum (https://github.com/jessievb/automated_CFA) was used to quantify Sall4-colony numbers automatically.

In-cell western, for colony counts

Samples were fixed with 4 % PFA for 15 minutes, followed by 0.3% triton X-100 permeabilization and 1 % BSA blocking for 30 minutes. After that, they were incubated with either SALL4 (Abcam; ab29112), SSEA1(R&D Systems; MAB-2155), or NANOG (eBioscience; 14-5761-80). Cells were washed twice with 1% PBS and stained with appropriate IRDye 800CW secondary antibodies (LI-COR) and counterstained with Draq5 (Thermo Scientific). As a staining control, a sample with only the secondary antibody was used to adjust the brightness and contrast properly, to avoid false-positives.

Images were acquired with an Odyssey CLx Infrared Imaging System (LI-COR) and Image Studio v5.0 software and adjusted for brightness and contrast with ImageJ and Adobe Photoshop CS6.

RNA isolation and RT-qPCR

Total RNA was isolated with Quick- RNA™ MicroPrep (Zymo Research) following the manufacturer´s instructions. RNA was eluted in RNAse-free water. Concentration was measured by absorbance with Nanodrop and we used Bioanalyzer (Agilent) to assess the quality and integrity of samples. cDNA was synthesized from 120-180 ng RNA with Superscript lll kit (Thermo Scientific). Each RT-qPCR reaction was done with 1-2 ng diluted cDNA and SYBR green mix (iQ-SYBR-Green Supermix, Biorad) and 10 µM primer pairs. Relative gene expression was calculated using the ΔΔCt method, using the housekeeping gene Gapdh as a reference.

CELseq2-RNAseq

The following adaptations were done to the original sample preparation: 100 pg purified RNA was directly added to a reverse transcription mixtures containing Maxima H Minus (ThermoFisher) reverse transcriptase and CELseq2 primers with a 6-nucleotide sample barcode and 8-nucleodide UMI. After reverse transcription samples were pooled and purified using AmpureXP beads (Beckman Coulter). Second strand synthesis and following steps were performed according to the original protocol.

The matrix with all the counts was analyzed with scater R package v. 1.3.49 (McCarthy et al., 2017) in order to assess the overall quality of the samples and to filter out those with low quality, based on default parameters. Scran v.1.6.9 (Lun et al., 2016) and scater R packages were used to correct for batch effects by regression, and also to normalize (log2-cpm).

Principal Component Analyses were conducted in scater and in gplots R packages. Most relevant Principal Components we found were PC1 and PC2. To analyze the most variable genes in the knockdowns (Figure 3B), the top 200 features (genes) correlating with either PC1 or PC2 were extracted. This set of genes was used for hierachical clustering based on the Pearson correlation of the knockdowns seen in Figure 3B.

Bulk RNA-seq, Gene Ontology and GSEA anaysis

For bulk RNA sequencing, Kapa-RNA HyperPrep kit with Ribo Erase was used for ribosomal depletion and library preparation (Roche, Kapa Biosystems), starting with 200 ng of total RNA. The libraries were amplified for 10 cycles, quantified with Qubit, checked for size distribution (300 bp) by Bioanalyzer (Agilent), and subjected to qPCR analysis before and after library preparation. Libraries were sequenced paired-end (Illumina NextSeq 500, read length 43 bp). Reads were aligned to the mouse genome (mm10) with STAR version 2.5.2b (Dobin et al., 2013).

For differential gene expression analysis, negative control was compared to the knockdowns using DESeq2 v.1.18.1 (Love et al., 2014). We filtered differential genes with a cutoff of log2-foldchange>1 and padj<0.05 as for gene ontology analysis with DAVID v.6.7 (Huang da et al., 2009).

For heatmap visualization and GSEA v.3.0, data log-cpm with EdgeR package v. 3.20.9 (Robinson et al., 2010).

All gene sets used for Gene Set Enrichment Analysis (Subramanian et al., 2005) , were obtained by process search in the Gene Ontology database. Normalized and low-count filtered RNA-seq expression datasets were used as an input for the initial enrichment analysis , which initially compared si*Wdr5* vs. control (Figure 5). The basic parameters used were 1000 gene-set permutations to calculate the enrichment score by the log2-ratio of classes metric.

Subsequently, leading edge analysis (Mootha et al., 2003) was performed for *Wdr5* vs control. Leading edge refers to the core genes contributing to the enrichment score. These genes were extracted from each of the sets (cell cycle checkpoint, DNA repair, DNA repair homologous recombination and cell adhesion). Those core genes were further used to determine the leading edge genes of either si*Brca1*, si*Bard1* or si*Wdr5* vs. control (Figure S5). The overlap of leading edge genes between the 3 knockdowns is what we have depicted in Figure 6C.

SUPPLEMENTAL REFERENCES

Dobin, A., Davis, C.A., Schlesinger, F., Drenkow, J., Zaleski, C., Jha, S., Batut, P., Chaisson, M., and Gingeras, T.R. (2013). STAR: ultrafast universal RNA-seq aligner. Bioinformatics 29, 15-21.

Huang da, W., Sherman, B.T., and Lempicki, R.A. (2009). Systematic and integrative analysis of large gene lists using DAVID bioinformatics resources. Nature protocols 4, 44-57.

Jones, T.R., Kang, I.H., Wheeler, D.B., Lindquist, R.A., Papallo, A., Sabatini, D.M., Golland, P., and Carpenter, A.E. (2008). CellProfiler Analyst: data exploration and analysis software for complex image-based screens. BMC bioinformatics 9, 482.

Love, M.I., Huber, W., and Anders, S. (2014). Moderated estimation of fold change and dispersion for RNA-seq data with DESeq2. Genome biology 15, 550.

Lun, A.T., McCarthy, D.J., and Marioni, J.C. (2016). A step-by-step workflow for low-level analysis of single-cell RNA-seq data with Bioconductor. F1000Research 5, 2122.

McCarthy, D.J., Campbell, K.R., Lun, A.T., and Wills, Q.F. (2017). Scater: pre-processing, quality control, normalization and visualization of single-cell RNA-seq data in R. Bioinformatics 33, 1179-1186.

Mikkelsen, T.S., Wakefield, M.J., Aken, B., Amemiya, C.T., Chang, J.L., Duke, S., Garber, M., Gentles, A.J., Goodstadt, L., Heger, A., et al. (2007). Genome of the marsupial Monodelphis domestica reveals innovation in non-coding sequences. Nature 447, 167-177.

Mootha, V.K., Lindgren, C.M., Eriksson, K.F., Subramanian, A., Sihag, S., Lehar, J., Puigserver, P., Carlsson, E., Ridderstrale, M., Laurila, E., et al. (2003). PGC-1alpha-responsive genes involved in oxidative phosphorylation are coordinately downregulated in human diabetes. Nature genetics 34, 267-273.

Pascual-Vargas, P., Cooper, S., Sero, J., Bousgouni, V., Arias-Garcia, M., and Bakal, C. (2017). RNAi screens for Rho GTPase regulators of cell shape and YAP/TAZ localisation in triple negative breast cancer. Scientific data 4, 170018.

Robinson, M.D., McCarthy, D.J., and Smyth, G.K. (2010). edgeR: a Bioconductor package for differential expression analysis of digital gene expression data. Bioinformatics 26, 139-140.

Subramanian, A., Tamayo, P., Mootha, V.K., Mukherjee, S., Ebert, B.L., Gillette, M.A., Paulovich, A., Pomeroy, S.L., Golub, T.R., Lander, E.S., et al. (2005). Gene set enrichment analysis: a knowledge-based approach for interpreting genome-wide expression profiles. Proceedings of the National Academy of Sciences of the United States of America 102, 15545-15550.
